# Editorial: From individual minds to language co-evolution: Psychological mechanisms for the evolution of cross-cultural and cross-species communication systems

**DOI:** 10.3389/fpsyg.2022.1040645

**Published:** 2022-10-06

**Authors:** Junru Wu, William S.-Y. Wang, Niels O. Schiller

**Affiliations:** ^1^Laboratory of Language Cognition and Evolution, Department of Chinese Language and Literature, East China Normal University, Shanghai, China; ^2^Leiden University Centre for Linguistics, Leiden, Netherlands; ^3^Department of Chinese and Bilingual Studies, Hong Kong Polytechnic University, Kowloon, Hong Kong SAR, China; ^4^Leiden Institute for Brain and Cognition, Leiden, Netherlands

**Keywords:** phylogeny, ontogeny, animal communication, language emergence, bilingualism, dialect, multimodal, lexical borrowing

“Co-evolution” was originally a term from ecology and referred to closely associated species influencing each other and leading to reciprocal changes (Thompson and Rafferty, 2020). Borrowing this term into language evolution, the contributors of the current Research Topic have not only included mutual influences of different biological species' communication systems, but also interactions of different linguistic varieties, as well as the adaptive modulation of different functional properties of linguistic systems. Eight articles have been published in this Research Topic, which can be divided into three sets according to the adopted approaches.

1. The first set of studies (four papers) focus on the comparison between humans and other species.

Two of the papers can be considered following the tradition of comparison between *Homo sapiens* and other primates (e.g., Premack and Premack, 1972) and shed light on the biological basis of human language emergence.

■ (1) *Faces and Voices Processing in Human and Primate Brains: Rhythmic and Multimodal Mechanisms Underlying the Evolution and Development of Speech* based on recent discovery of “a third visual pathway,” reviewed empirical studies addressing the processing of visual orofacial cues and its integration with vocal auditory cues and discussed how evolution and development of a rhythmic and multimodal organization of sensorimotor brain networks could contribute to the emergence and evolution of human speech (Michon et al.).
■ (2) *Evidence of Grammatical Knowledge in Apes: An Analysis of Kanzi's Performance on Reversible Sentences* re-investigated the bonobo's comprehension of reversible sentences (e.g., “Pour the Coke in the lemonade” vs. “Pour the lemonade in the Coke.”). Unlike earlier claims, Kanzi's performance actually vastly exceeds random chance, suggesting that *non-human primates can also understand word order grammatical rules*, which may be attributed to homologous instead of divergent brain circuitry (Schoenemann).
■ This study on the bonobo Kanzi can be weighed alongside with a human study in this collection (3) *Investigating Word Order Emergence: Constraints From Cognition and Communication*, which also investigated reversibility, but focused on human gesture production of reversible and irreversible events (Schouwstra et al.). It is known from previous studies that in gesturing the word order does not just follow the speakers' native spoken languages (e.g., Gibson et al., 2013). This experimental study showed that the variability in word order depends on properties of both the verb and the direct object. The authors explained the findings with the “noisy channel” account from information theory. Interestingly, gesture orders of human individuals get more consistent over time, which may imply a strategy-based explanation for word order emergence.

The fourth paper investigated less related but closely associated species and explored the influence of common cultural selection procedure.

■ (4) *Did Dog Domestication Contribute to Language Evolution?* based on a comprehensive review of biological, neurological, and archaeological studies, raised *the hypothesis of a parallel human-canine domestication:* in the reciprocal influences between the two species, selected controlling of reactive aggression and prosocial behavior may have provided the critical cultural and physiological bases for more complex forms of human languages to emerge (Benítez-Burraco et al.).

2.The second set of studies (three papers) investigated human individuals who use two linguistic varieties. These papers may shed light on how microscopic changes happening in bilingual and bi-dialectal minds collectively modulate the evolutionary process of co-evolving languages and dialects.

Two of the papers investigated the influences of bilingual or bi-dialectal experiences on cross-linguistic behaviors.

■ (5) *Different Neural Responses for Unfinished Sentence as a Conventional Indirect Refusal Between Native and Non-native Speakers: An Event-Related Potential Study* investigated the pragmatic processing of conventions interculturally (Wang et al.). The authors identified event-related potential (ERP) components related to increased pragmatic processing load induced by the non-nativeness of the language. Interestingly, the ERP responses were also found to be modulated by individual mentalizing ability, in line with the above-mentioned inter-species studies (4).
■ (6) *Effects of Familiarity and Dialect Experience on the Description of Tonal Variant* found that variation of sociophonetic factors across Chinese tonal dialects can predict the bi-dialectal listeners' descriptive decisions on tone height variants, and suggested that individuals' cross-dialectal perception may influence cultural coevolution of cross-dialect tonal variation (Liu and Gibbon).

Different from these two papers and previous studies on bilingualism, the third paper directly tapped into the process of linguistic changes happening in individual minds.

■ (7) *Cross-Dialectal Novel Word Learning and Borrowing* provided the first experimental evidence that monolectals and bi-dialectals adopt different mechanisms and differ in abilities in borrowing words (Wu et al.). These experimental findings were discussed with historical linguistic evidence to suggest that bi-dialectism as a demographic feature may shape the route of lexical evolution of co-evolving linguistic varieties.

3. While the previous two approaches focus on the role of individuals in language co-evolution, the last study (one article) treats languages as self-organizing systems and investigated the interaction of functional constraints and evolutionary mechanisms in language systems.

■ (8) *Structural Variability Shows Power-Law Based Organization of Vowel Systems* conducted cross-linguistic typological and phylogenetic analyses on the worldwide phonetic database of vowel systems (Becker-Kristal, 2010) and revealed a power-law based dependence between the *global structural dispersion* and the *local focalization* mechanisms of vowel systems (Zhang and Gong). The findings illustrated that correlated evolutions of these two mechanisms may proceed in an adaptive process and evolutionarily shaped the vowel systems in the world's languages.

Taken together, this collection of papers provided multiple views and efforts from various angles to entangle the complexity in the evolution of languages. Although each paper probably has only broken a tip of this mountainous iceberg, we may still revisit the biological metaphor of “co-evolution” introduced at the beginning of this Research Topic: comparing languages to genomes, and linguistic communities to species. While embracing this metaphor, we hope our collection can also bring more attention to the fact that language is still different from genomes as a cultural phenomenon bore in individuals' minds and collective behaviors. The “co-evolution” of languages thus involves unique cognitive mechanisms as compared with the co-evolution of species, which is still calling for further inquiry.

## Author contributions

JW drafted the original version of this editorial. WW and NS revised the manuscript multiple times. All authors contributed to the article and approved the submitted version.

## Funding

JW's work was supported by the Chinese Fundamental Research Funds for the Central Universities (2017ECNU-YYJ017), by Shanghai Philosophy and Social Sciences Fund (2017BYY001), and by National Social Science Fund Major Projects (18ZDA296).

## Conflict of interest

The authors declare that the research was conducted in the absence of any commercial or financial relationships that could be construed as a potential conflict of interest.

## Publisher's note

All claims expressed in this article are solely those of the authors and do not necessarily represent those of their affiliated organizations, or those of the publisher, the editors and the reviewers. Any product that may be evaluated in this article, or claim that may be made by its manufacturer, is not guaranteed or endorsed by the publisher.
